# Increased D-dimer level was a poor predictor of neuroblastoma, especially in the high-risk group

**DOI:** 10.1186/s12957-023-02974-2

**Published:** 2023-03-14

**Authors:** Yue Ma, Changchun Li, Zhenzhen Zhao, Chao Yang, Jianwu Zhou, Liang Peng, Xiaobin Deng, Shan Wang

**Affiliations:** Department of Pediatric Surgical Oncology, Ministry of Education Key Laboratory of Child Development and Disorders, National Clinical Research Center for Child Health and Disorders, China International Science and Technology Cooperation base of Child Development and Critical Disorders, Chongqing Key Laboratory of Pediatrics, Children’s Medical Big Data Intelligent Application Chongqing University Engineering Research Center, Children’s Hospital of Chongqing Medical University, Chongqing, 400014 People’s Republic of China

**Keywords:** D-dimer, Neuroblastoma, Prognosis, Risk stratification

## Abstract

**Purpose:**

D-dimer levels are associated with tumor progression and prognosis in various cancers. However, there are few research about the relationship between D-dimer and neuroblastoma (NB). This study assessed the relationships of D-dimer levels with clinical features and overall survival (OS) in patients with NB.

**Methods:**

Information about the clinical features of 365 patients and the prognosis of 301 patients was collected. The relationship between D-dimer levels and clinical features or OS was analyzed. We constructed the risk score based on Cox regression analysis and verified the predictive efficacy of the model through ROC curve and calibration curve.

**Results:**

The results showed that D-dimer levels were significantly increased in patients with nonmediastinal tumor, tumor larger than 10 cm, stage 3–4 disease, bone marrow metastasis, unfavorable histology, bone metastasis, *NMYC* amplification, and the high-risk group (all *P* < 0.05). The Kaplan–Meier survival analysis showed that there were significant differences in 3- and 5-year OS (87.4% vs. 32.3%, 79.3% vs. 32.3%, *P* < 0.0001) between the low D-dimer and high D-dimer groups. In the high-risk group, the OS of high D-dimer was significantly lower than that of low D-dimer (*P* < 0.0001). All cases were divided into the training cohort (*N* = 211) and the validation cohort (*N* = 90). Multivariate analysis further suggested that D-dimer level, bone metastasis, and *NMYC* status were independent prognostic factors for OS (all *P* < 0.05). Based on the above three factors, we constructed the risk score in the training cohort. Survival analysis showed that compared with the other groups, the group with 11 scores had the worst prognosis (3-year OS 0%, *P* < 0.0001). The time-dependent ROC analysis and calibration curve indicated that the risk score had good accuracy.

**Conclusions:**

Patients with high D-dimer levels tended to have unfavorable clinical characteristics and poor prognosis.

## Introduction

D-dimer is the smallest fragment of the degradation product of fibrous protein and a valuable biomarker of thrombin formation and fibrinolysis [1, 2]. The increased D-dimer level is associated with a hypercoagulable state and secondary hyperfibrinolysis in the body [3]. In pathological conditions, the concentrations of D-dimer can increase, such as injury, cancer, or infections [4, 5]. In emergency medicine, a negative D-dimer test is valuable to exclude the diagnosis of pulmonary embolism [6]. In orthopedics, patients with high D-dimer levels during knee arthroplasty may be more likely to be diagnosed with periprosthetic joint infection after the operation [7]. D-dimer levels are not only important in disease diagnosis but also negatively correlated with prognosis in many diseases. For example, the fatality rate of patients with COVID-19 is higher in the high D-dimer level group than in the low D-dimer level group [8]. Metastatic colorectal cancer patients with elevated D-dimer have significantly poorer overall survival (OS) and progression-free survival [9]. Moreover, elevated D-dimer levels suggest a shorter survival time in lung cancer patients [10, 11]. Coagulation activation has been found in many cancer patients. Coagulation activation is considered as an important promoting factor for tumor dissemination and growth. The hypercoagulable state of patients with malignant tumors is considered to be related to tumor metastasis and poor prognosis [12]. Moreover, coagulation activation promotes tumor cell extravasation and tumor metastasis [13].

Neuroblastoma (NB) is a common pediatric tumor with high incidence and mortality. The heterogeneity of NB leads to a series of clinical and biological indicators for prognosis prediction, such as age, tumor stage, and *NMYC*. Treatment of NB depends on the individual involved, and accuracy in clinical staging and risk stratification is extremely vital for planning therapeutic methods and prognosis. The therapy for the low-risk group is safe and feasible, while the treatment of high-risk NB includes surgery, chemotherapy, radiation, hematopoietic stem cell transplantation, immunotherapy, and targeted therapy [14, 15]. Even after multimodal therapy, the 5-year OS for high-risk NB is still below 50% [16]. So we need to find new biomarkers to further stratify the risk of NB, especially in the high-risk group, to improve the prognosis of children.

However, we find few studies analyzing the correlation between D-dimer levels and NB. Chen et al [17] retrospectively explored the clinical information of 81 children with NB and found that elevated D-dimer levels were significantly associated with advanced disease stage and *NMYC* amplification. We hypothesize that D-dimer, as a common clinical indicator, may be helpful for the risk classification of NB. In this study, we explored correlations between D-dimer level and clinical features or prognosis of children with NB and combined with clinical information and laboratory examinations to find a new risk stratification indicator in NB.

## Materials and methods

### Patients

Information about the baseline clinical and prognostic conditions of 365 patients with NB between June 2012 and December 2020 in Children's Hospital of Chongqing Medical University was collected and retrospectively reviewed. Patients were diagnosed by two methods: one is the pathological examination and the other one is bone marrow cytological examination and assessment of the levels of catecholamines and their metabolites. The second method is suitable for critical patients who cannot bear the risk of biopsy operation or parents who require treatment without pathological examination. Patients with trauma, immunological, infection, hemostatic or thrombotic disorders, or those under anticoagulant treatment were excluded. Tumor stage and risk classification of patients were based on the International Neuroblastoma Staging System (INSS) and the Children’s Oncology Group (COG) risk classification system, respectively. The survival time started from the date of diagnosis and ended on the date of death or the last follow-up date, and the deadline date of follow-up was March 31, 2021.

### Treatment

The treatment plan was based on the COG risk classification system. The commonly used chemotherapy drugs in our department included cyclophosphamide, vincristine, cisplatin, etoposide, adriamycin, etc. The low-risk group received surgery after hospitalization, and whether to receive chemotherapy or not was based on individual conditions. Whether or not the intermediate-risk group received radiotherapy after surgery and chemotherapy depended on the patient's condition. The high-risk group received comprehensive treatment, including surgery, adjuvant chemotherapy, radiotherapy, 13-cis-retinoic acid, and traditional Chinese medicine.

### Laboratory testing

All patients were in the hospital, and blood was drawn within 24 h of hospitalization. Venous blood was extracted before treatment. The D-dimer test was performed in our hospital’s laboratory. D-dimer (mg/L) was measured by immunoturbidimetric assay with the Sysmex CA-1500 automated coagulation instrument (Sysmex Corporation, Kobe, Japan). D-dimer > 0.55 mg/L was considered abnormal. *NMYC* status was determined by FISH or next-generation sequencing.

### Statistical analysis

The primary endpoint was OS time, defined as the time from diagnosis to death, or last follow-up if the patient was alive. Tables, figures, and statistical analysis were conducted by using SPSS software (21.0) and R software (3.6.2). The Mann–Whitney *U* test was used to examine the association between D-dimer level and clinical features such as age, sex, tumor size, pathological classification, and metastasis. The Kaplan–Meier method was used to analyze the possibility of OS by variable, and the log-rank test was performed to calculate the significant differences between groups. Those factors with *P* < 0.1 in univariate regression analyses were included in the multivariate Cox regression analysis to further examine the risk factors for OS. A two-sided *P* < 0.05 was considered as statistically significant difference.

The 95% confidence intervals (CIs) for the hazard ratio (HR) and *P* were given together according to multivariate Cox regression analysis. Based on the HR, integer points were derived that form the basis of an additive scoring system. The time-dependent receiver operator characteristic (ROC) curve and calibration curve were used to compare the predictive accuracy of the risk score in the training cohort and validation cohort.

## Results

### Baseline characteristics

The clinical characteristics of 365 NB patients were retrospectively collected and evaluated. The baseline clinical characteristics of patients with NB were presented in Table 1. Among these 365 patients, 212 were males and 153 were females. The median age at the initial examination was 29 months (range of 0.1–156 months), and the median follow-up time was 22.5 months (range of 1–115 months). The median D-dimer level in the initial examination was 1.09 mg/L (range 0–97.02 mg/L).

**Table 1:** Baseline clinical characteristics of 365 patients with NB

Variable	*N*	%	D-dimer median (mg/L)	*P*
Gender				
Male	212	58.08	1.64	0.427
Female	153	41.92	0.89	
Age (months)				
≤ 18	118	32.33	0.80	0.002
> 18	247	67.67	2.24	
Tumor site				
Mediastinal tumor	69	18.90	0.35	< 0.001
Other position tumor	296	81.10	1.52	
Pathology classification				
FH	147	44.55	0.47	< 0.001
UFH	183	55.45	2.85	
Unknown	35	/		
Bone marrow metastasis				
Yes	130	35.62	3.83	< 0.001
No	235	64.38	0.56	
Bone metastasis				
Yes	103	29.60	4.00	< 0.001
No	245	70.40	0.75	
Unknown	17	/		
*NMYC* status				
Amplified	68	20.80	6.00	< 0.001
Not amplified	259	79.20	0.91	
Unknown	38	/		
INSS stage				
1,2,4s	64	17.53	0.34	< 0.001
3,4	301	82.47	1.64	
COG risk classification				
Non-high risk	142	38.90	0.42	< 0.001
High risk	223	61.10	2.88	

*FH* favorable histology, *UFH* unfavorable histology, *INSS* International Neuroblastoma Staging System

The association between D-dimer level and clinical features among NB patients was also shown in Table 1. The results showed that D-dimer levels were significantly increased in patients with age above 18 months, non-mediastinal tumor, bone marrow metastasis, unfavorable histology (UFH), bone metastasis, *NMYC* amplification, INSS stage 3–4, and high-risk group (all *P* < 0.05). However, no significant difference was found between D-dimer level and gender (*P* > 0.05).

### D-dimer level in the prediction of prognosis for NB

**Fig. 1:**
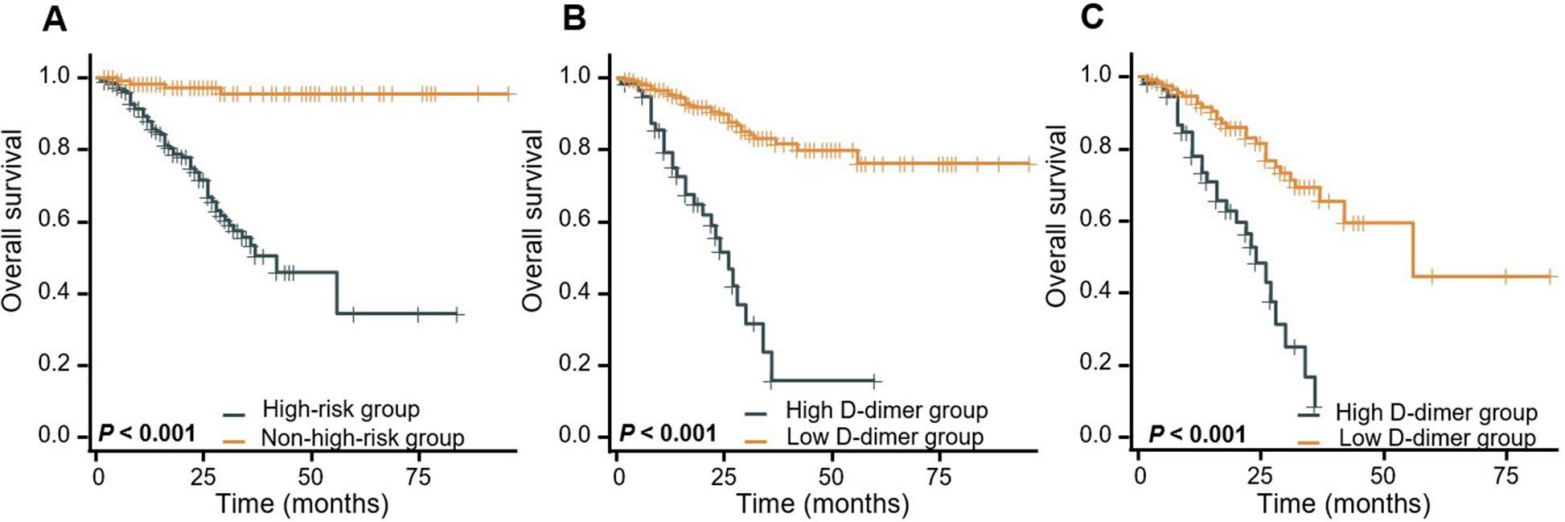
Kaplan–Meier analysis of patients with NB. **A** High-risk group VS. non-high-risk group. **B** High D-dimer group VS. low D-dimer group. **C** Kaplan-Meier analysis in subgroups of high-risk group

Among the 365 children, 64 children quitted treatment or missed some examination results, so survival analysis was carried out for 301 patients. The Kaplan–Meier survival analysis showed that there were significant differences in 3- and 5-year OS (95.6% vs. 53.4%, 95.6% vs. 34.4%, *P* < 0.001) between the non-high-risk group and the high-risk group (Fig. 1A). Moreover, the level of D-dimer is a continuous variable, so we used R software to determine the optimal cutoff value (5.2 mg/L) of the D-dimer level. According to the optimal cutoff value, 301 patients were classified into the high D-dimer group and the low D-dimer group. The numbers of patients in the low D-dimer group and high D-dimer group were 58 and 243, respectively. The Kaplan–Meier survival analysis showed that there were significant differences in 3- and 5-year OS (87.4% vs. 32.3%, 79.3% vs. 32.3%, *P* < 0.0001) between the low D-dimer and high D-dimer groups (Fig. 1B).

To further explore the value of D-dimer in prognosis prediction, we divided the high-risk group into two subgroups. There were 120 low D-dimer and 55 high D-dimer in the high-risk group. As shown in Fig. 1C, in the high-risk group, the OS rate of the low D-dimer group was significantly better than that of the high D-dimer group (*P* < 0.001).

### Multivariable model and risk score

**Fig. 2:**
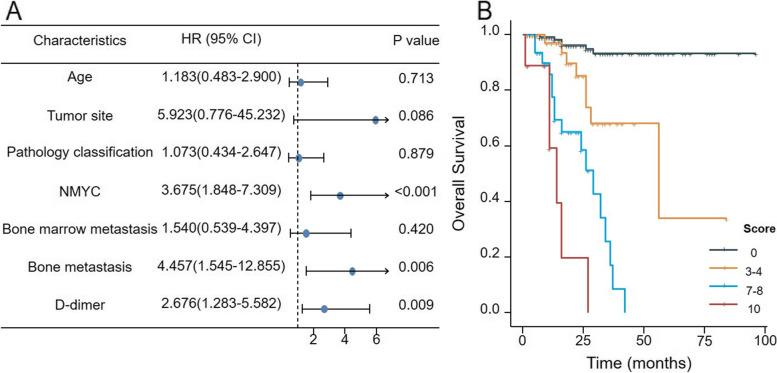
**A** Forest plot of HR (with 95% CI) from univariate Cox models of OS (*N* = 211). Integer risk scores of D-dimer level, bone metastasis and NMYC status were defined by HR. D-dimer > 5.2 mg/L was assigned score of 3, bone metastasis was assigned score of 4 and NMYC amplified was assigned score of 4. **B** Survival curves of patients with different risk scores

Univariate and multivariate analyses were performed to explore the factors that influenced the prognosis of patients with NB. All cases were divided into training cohorts (*N* = 211) and validation cohorts (*N* = 90). In the training cohort, univariate analysis showed that age above 18 months, non-mediastinal tumor, UFH, INSS stage 3–4, bone marrow metastasis, bone metastasis, *NMYC* amplification, high-risk group, and high D-dimer group were unfavorable factors for OS (all *P* < 0.05) (Table 2). Moreover, the multivariate analysis further suggested that D-dimer level, bone metastasis, and *NMYC* status were independent prognostic factors for OS (all *P* < 0.05) (Fig. 2A). Integer risk scores of D-dimer level, bone metastasis, and *NMYC* status were defined by HR. The HR for D-dimer > 5.2 mg/L was 2.676, assigned a score of 3; the HR for bone metastasis was 4.457, assigned a score of 4; and the HR for *NMYC* amplification was 3.675, assigned a score of 4. In Fig. 2B, the 211 patients were divided into four groups based on risk scores. Survival analysis showed that the group with the highest scores had the worst prognosis. The 3-year OS was 93.4% for score = 0 versus 0% for score = 11 (*P* < 0.0001).

**Table 2:** Univariate analysis of OS

	Univariate analysis	
	HR (95% CI)	*P*
Age	2.732 (1.241–6.015)	0.013
Tumor site	0.103 (0.014–0.752)	0.025
D-dimer	8.205 (4.085–16.481)	< 0.001
Pathology classification	4.377 (1.978–9.683)	< 0.001
Bone marrow metastasis	6.551 (3.203–13.399)	< 0.001
Bone metastasis	11.610 (5.530–24.372)	< 0.001
*NMYC* status	6.727 (3.505–12.910)	< 0.001
INSS stage	10.056 (1.378–73.389)	0.023
COG risk classification	23.481 (5.570–98.987)	< 0.001

### Verification of risk score

**Fig. 3:**
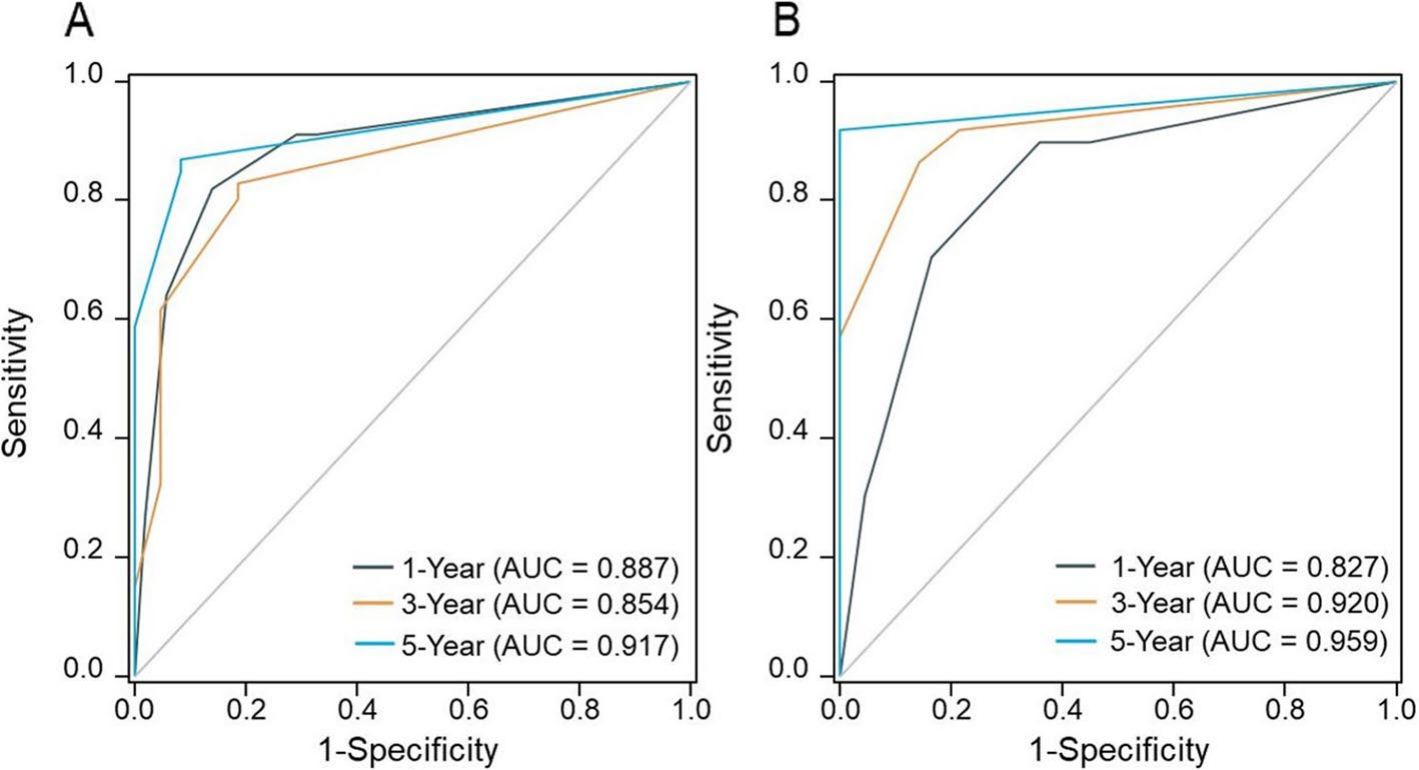
**A** ROC curves for discrimination of training cohort. **B** ROC curves for discrimination of validation cohort

**Fig. 4:**
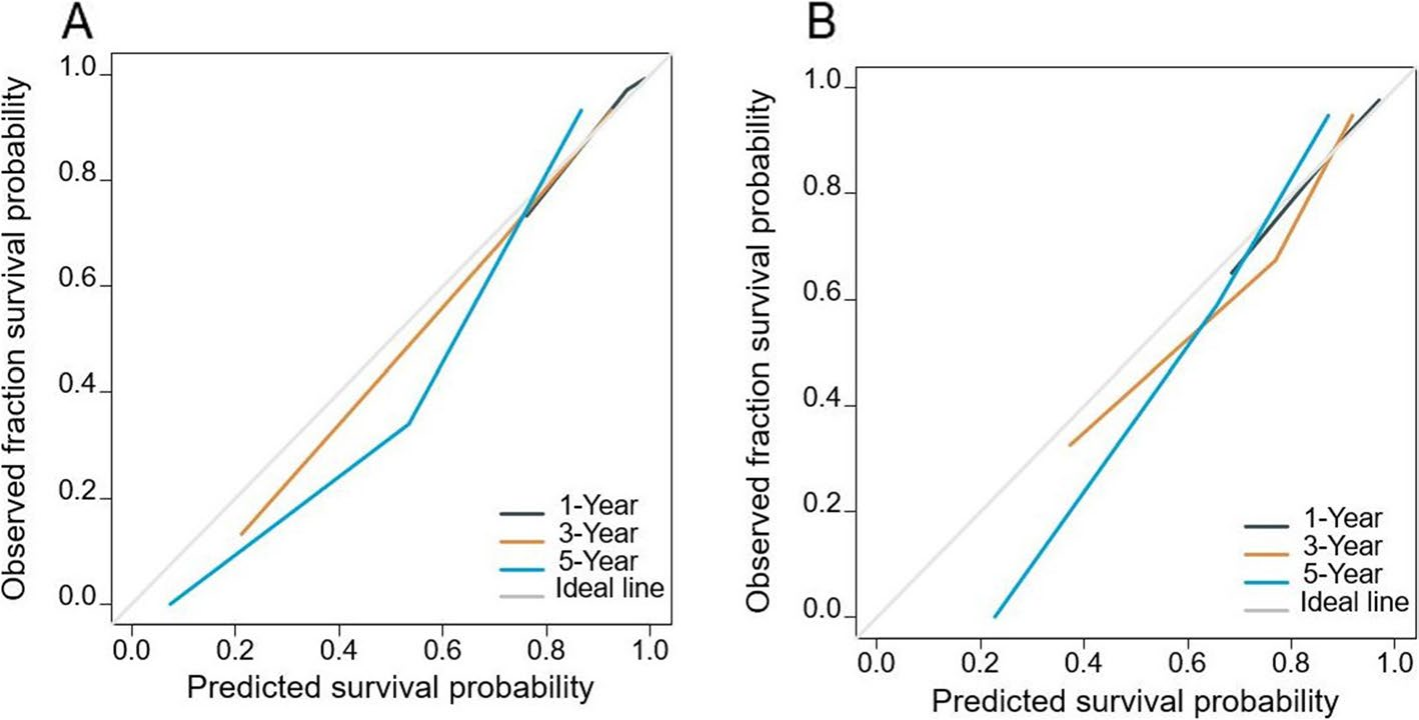
**A** Evaluation of calibration plots based on OS of training cohort in 1, 3, and 5 years. **B** Evaluation of calibration plots based on OS of validation cohort in 1, 3, and 5 years. The slanted gray line represents an ideal match between the actual survival (*y*-axis) and risk score predicted survival (*x*-axis). The perpendicular line means 95% CIs. Closer distances from the points to the dashed line indicate higher prediction accuracy

The performance of the test was assessed using time-dependent ROC analysis for 1-year, 3-year, and 5-year OS. The area under the curve (AUC) values of the training cohort were 0.887, 0.854, and 0.917, respectively (Fig. 3A). The validation cohort's AUC values were 0.827, 0.920, and 0.959, respectively (Fig. 3B). The AUC value indicated that the prognostic accuracy of the risk score was good. Moreover, the calibration curves of the training cohort and validation cohort were shown in Fig. 4. The calibration curve showed that the 1-year survival rate prediction effect was good and the prediction of the 3-year and 5-year survival rate by the risk score underestimate the actual risk in the low-risk population. In summary, the prediction curve and the ideal curve in the calibration diagrams of the risk score fitted well, indicating that the risk score had a good degree of calibration.

## Discussion

The treatment of NB is based on the risk classification of patients. Even after complex treatment, the survival rate of high-risk NB patients has not been satisfactory, so it is urgent to find new risk classification indicators and treatment methods. Accurate risk classification can help develop treatment plans in line with the patient’s condition and reduce the possibility of insufficient treatment of high-risk patients and overtreatment of low-risk patients. D-dimer may provide useful information to predict prognosis in patients with solid tumors [18]. High D-dimer level has been reported to be closely related to the poor prognosis of other tumors, such as nasopharyngeal cancer [19], gastric cancer [20], and cholangiocarcinoma [21]. However, we found few studies analyzing the correlation between D-dimer levels and clinical features or prognosis for patients with NB. D-dimer may predict the prognosis of children with NB. D-dimer tests are easy, rapid, and cost-effective in clinical settings, which will be smoothly finished even in primary hospital pediatric clinics. Thus, the importance of the D-dimer level for NB is unneglectable.

In our study, high D-dimer levels in NB were significantly associated with factors raising the risk of a poor outcome, such as huge tumor, amplified *NMYC*, and stage 3–4. Patients with high D-dimer level had lower survival rates both in NB patients and in the high-risk group. Multivariate analysis further suggested that the D-dimer level, bone metastasis, and *NMYC* status were independent prognostic factors for OS (all *P* < 0.05). Based on the above three factors, we constructed the risk score and compared the accuracy of the risk score in prognostic prediction. The time-dependent ROC analysis and calibration curve indicated that the risk score had good accuracy. All of these results suggested that high D-dimer level may be an unfavorable prognostic predictor for NB.

Malignancy and the hemostatic system may mutually interfere to promote disease progression [22, 23]. Basic research indicates that hemostatic components are closely associated with cancer biology in multiple ways [24]. Tumor cells can promote the body to secrete more tissue factor (TF) which is the primary initiator of the coagulation cascade, and procoagulant substances, and interact with vascular endothelial cells, platelets, and monocytes, resulting in a hypercoagulable state [25]. And hypercoagulable state in the blood is related to the development and metastasis of cancer [26, 27]. The growth and dissemination of tumor requires rapid angiogenesis, while the coagulation cascade can be activated [28]. Tumor angiogenesis is mainly mediated by TF-induced thrombin production and subsequent cross-linked fibrin deposition and cross-linked fibrin provides a temporary angiogenesis matrix to promote vascular invasion [29]. Moreover, Tumor activated platelets further promote the progress of cancer by promoting angiogenesis and tomor metastasis [30]. Tumor metastasis further contributes to the generation and lysis of fibrin, increasing the level of D-dimer which level correlates with tumor burden and the number of metastases [24].

TF is the main promoter of blood coagulation and the regulator of cancer angiogenesis. According to the report, there was a significant association between TF expression and NB prognosis [31]. What is more, the situation of the balance of angiogenesis in neuroblastoma is complex, and a series of molecules related to angiogenesis are detected in neuroblastoma, such as vascular endothelial growth factor, IL-8, fibroblast growth factor 2 [32, 33]. That showed coagulation system plays an important role in the occurrence and development of NB. D-dimer level is an index reflecting the coagulation function of the body. The D-dimer test is easily finished, reproducible, and low cost, which may be a potential predictor to be used in NB risk stratification. In our study, the D-dimer levels of patients with bone marrow metastasis and bone metastasis were significantly higher than those without metastasis. The mechanism may be that the coagulation system of NB is activated, the formation of fibrin is increased, and the adhesion between platelets and tumor cells is enhanced, which is conducive to the proliferation and invasion of tumor cells. Advanced-stage and high-risk group patients were more likely to develop elevated D-dimer levels. The above results show that the increased D-dimer level may play an important role in predicting tumor metastasis and disease progression for NB patients. Age, stage of disease, *NMYC* status, pathology classification, and histology are regarded as important risk factors to predict prognosis for NB [34]. Correlation analysis showed that non-mediastinal tumor, tumor more than 10 cm, tumor metastasis, UFH, and *NMYC* amplification, which predict poor prognosis for NB, were all related to the increase of D-dimer level. The D-dimer level is significantly different among NB patients in different groups and is closely related to the clinical characteristics of NB patients.

High D-dimer levels are associated with poor prognosis and increased mortality risk in cancer patients [35]. D-dimer is also valuable in predicting an augmented risk of cancer recurrence [36]. In our study, the Kaplan–Meier survival analysis showed that the increased level of D-dimer suggested a significant increase in mortality for NB. The survival analysis of subgroups in the high-risk group showed the significance of D-dimer in prognosis prediction. Multivariate analysis further suggested that D-dimer levels were independent prognostic factors for OS. According to the risk score we constructed, D-dimer > 5.2 mg/L was assigned a score of 3; bone metastasis was assigned a score of 4; and NMYC amplification was assigned a score of 4. Patients with 11 scores had the worst prognosis. The risk score has good predictive efficiency and can be used to assist clinical diagnosis and prediction, which may be promoted in the majority of primary hospitals.

## Limitation

Moreover, the limitations of our study must be mentioned. External validation of the risk score was lacking. Our study is a retrospective study at a single clinical center. To further verify the accuracy of our conclusions, it is necessary to conduct a prospective study with data from multiple centers.

## Conclusion

In summary, D-dimer was identified as an independent prognostic factor for NB patients. Patients with high D-dimer levels tended to have unfavorable clinical characteristics and poor prognoses. The risk score may be considered to further classify the risk group, which could provide a new index for the risk classification of NB.

## Data Availability

Data are available within the article. Other data are available from the corresponding authors on reasonable request.

## Abbreviations

AUC: Area under the curve
CI: Confidence intervals
COG: Children's Oncology Group
FH: Favorable histology
HR: Hazard ratio
INSS: International Neuroblastoma Staging System
NB: Neuroblastoma
OS: Overall survival
ROC: Receiver operator characteristic
UFH: Unfavorable histology
